# Supplementary material for: Semi-supervised Bayesian integration of multiple spatial proteomics datasets
Source: PLoS Comput Biol. 2025 Dec 15;21(12):e1013799. doi: 10.1371/journal.pcbi.1013799 (PMC12721539; doi:10.1371/journal.pcbi.1013799)
Supplement: S1 Text — Model and computational derivations (Section A), simulation study descriptions (Section B), and additional model convergence analyses for the Toxoplasma gondii application (Section C). Includes supporting figures (A, B) and additional references. (PDF) [file pcbi.1013799.s001.pdf]

Supplement: Semi-supervised Bayesian integration of multiple spatial proteomics datasets

Stephen Coleman[1,*], Lisa Breckels[2], Ross F. Waller[2], Kathryn S. Lilley[2], Chris Wallace[1,3,4], Oliver M. Crook[5,6,‡], Paul D.W. Kirk[1,3,4,‡]

1 MRC Biostatistics Unit, University of Cambridge, Cambridge, UK

2 Department of Biochemistry, University of Cambridge, Cambridge, UK

3 Department of Medicine, University of Cambridge, Cambridge, UK

4 Cambridge Institute of Therapeutic Immunology & Infectious Disease, University of Cambridge, Cambridge, UK

5 Department of Chemistry, University of Oxford, Oxford, UK

6 Kavli Institute for Nanoscience Discovery, University of Oxford, Oxford, UK

* stephen.d.p.coleman@gmail.com

‡ These authors contributed equally to this work.

A Derivations

A.1 Gaussian process mixture models

If we consider the mean vector of a Gaussian density as the finite realisation of an infinite function in our feature space, we can place a Gaussian process prior on it. Thus, for the kth component of this mixture, mean vector μ_k and observed data which has been transformed to a vector, $X_k = [X_{k_1}, \ldots, X_{k_{N_k}}]$,

$$X_k|\mu_k, \sigma_k \sim \mathcal{N}(\mu_k, \sigma_k^2 I_P), \tag{1}$$

$$\mu_k|a_k, l_k \sim GP(0, C_k). \tag{2}$$

As we assume exchangeable data for ease of notation we permute the original dataset such that we can denote X_k with contiguous indices, i.e., $X_k = [X_1, \ldots, X_{N_k}]$. The posterior distribution can be derived by using the properties of the multivariate Gaussian relating to conditional distribution of two jointly distributed

Gaussian variables [1],

$$\begin{bmatrix} y \\ f(X^*) \end{bmatrix} \sim \mathcal{N}\left(\mathbf{0}, \begin{bmatrix} C(X,X)+\sigma^2\mathbf{I} & C(X,X^*) \\ C(X,X^*) & C(X^*,X^*) \end{bmatrix}\right), \tag{3}$$

$$\implies \begin{cases} \mathbb{E}(f(X^*)|y) & = C(X^*,X)(C(X,X)+\sigma^2\mathbf{I})^{-1}y, \\ \mathbb{C}ov(f(X^*)|y) & = C(X^*,X^*) - C(X^*,X)(C(X,X)+\sigma^2\mathbf{I})^{-1}C(X,X^*). \end{cases} \tag{4}$$

We can then say

$$(\mu_k|X_k,\sigma_k,a_k,l_k) \sim GP(m_k,\tilde{C}_k), \tag{5}$$

$$m_k = C_k[1:P](C_k+\sigma_k^2 I_{N_K P})^{-1}X_k, \tag{6}$$

$$\tilde{C}_k = C_k[1:P,1:P] - C_k[1:P,](C_k+\sigma_k^2 I_{N_K P})^{-1}C_k[,1:P]. \tag{7}$$

Note that this involves taking the inverse of an $N_k P \times N_k P$ matrix, which is frequently a very costly calculation. There has been much exploration of scaling GP models such as sparse approximations [2] and low-dimensional approximations [3], see [4] for a recent review of the subject. However, in our application we have rich structure in the data as all items within a view have the same time/spatial measurements with consistent distance between each measurement and we can use this to reduce the computational load without using any of these more complex methods [5].

Let A_k be the $P \times P$ matrix defined by the squared exponential kernel function for the P measurements of a single observation, i.e.,

$$(A_k)_{ij} = a_k^2 \exp\left\{-\frac{d(t_i,t_j)^2}{2l_k^2}\right\},\ i,j = \{1,\ldots,P\}, \tag{8}$$

then A_k is positive symmetric and Toeplitz [6] and

$$C_k+\sigma_k^2 I_{N_K P} = \begin{bmatrix} A_k+\sigma_k^2 I_P & A_k & \cdots & A_k \\ A_k & A_k+\sigma_k^2 I_P & \cdots & A_k \\ \vdots & \vdots & \ddots & \vdots \\ A_k & A_k & \cdots & A_k+\sigma_k^2 I_P \end{bmatrix}. \tag{9}$$

As [7] have shown, we can write

$$C_k = J_{N_k} \otimes A_k \tag{10}$$

where J_{N_k} is $N_k \times N_k$ matrix of ones and $\otimes$ is the Kronecker product. Following the derivation from the supplement of [7], by letting $Q_k = I_P + \sigma_k^{-2} N_k A_k$ we can write

$$(C_k + \sigma_k^2 I_{N_K P})^{-1} = \frac{1}{\sigma_k^2} I_{N_k P} - \frac{1}{N_k \sigma_k^2} J_{N_k} \otimes (I_P - Q^{-1}). \tag{11}$$

Thus only a $P \times P$ matrix has to be inverted. Writing this out explicitly:

$$(C_k + \sigma_k^2 I_{N_K P})^{-1} =$$
$$\frac{1}{\sigma_k^2} \begin{bmatrix} I_P - \frac{1}{N_k}(I_P - Q^{-1}) & -\frac{1}{N_k}(I_P - Q^{-1}) & \cdots & -\frac{1}{N_k}(I_P - Q^{-1}) \\ -\frac{1}{N_k}(I_P - Q^{-1}) & I_P - \frac{1}{N_k}(I_P - Q^{-1}) & \cdots & -\frac{1}{N_k}(I_P - Q^{-1}) \\ \vdots & \vdots & \ddots & \vdots \\ -\frac{1}{N_k}(I_P - Q^{-1}) & -\frac{1}{N_k}(I_P - Q^{-1}) & \cdots & I_P - \frac{1}{N_k}(I_P - Q^{-1}) \end{bmatrix} \tag{12}$$

By noticing that each block column consists of a single occurrence of the identity matrix and N_k occurrences of $-\frac{1}{N_k}(I_P - Q^{-1})$, and this object only occurs when multiplied by C_k, a matrix consisting of N_k repeated blocks of A_k, we can simplify the object of interest to

$$C_k[1:P,](C_k + \sigma_k^2 I_{N_K P})^{-1} =$$
$$\frac{1}{\sigma_k^2} \begin{bmatrix} (A_k - A_k \times (I_p - Q^{-1})) & \cdots & (A_k - A_k \times (I_p - Q^{-1})) \end{bmatrix}. \tag{13}$$

This object occurs in both the posterior covariance function and the posterior mean function. In the latter,

$$C_k[1:P,](C_k + \sigma_k^2 I_{N_K P})^{-1} X_k =$$
$$\frac{1}{\sigma_k^2} \begin{bmatrix} (A_k - A_k \times (I_p - Q^{-1})) & \cdots & (A_k - A_k \times (I_p - Q^{-1})) \end{bmatrix} X_k \tag{14}$$
$$= \frac{1}{\sigma_k^2}(A_k - A_k \times (I_p - Q^{-1}))(X_1 + X_2 + \cdots + X_{N_k}) \tag{15}$$
$$= \frac{N_k}{\sigma_k^2}(A_k - A_k \times (I_p - Q^{-1}))\bar{X}_k, \tag{16}$$

where $\bar{X}_k$ is the sample mean of the component data.

For the final product of $C_k[1 : P,](C_k + \sigma_k^2 I_{N_K P})^{-1} C_k[,1 : P]$, we note that we have reduced the

multiplication of the first two objects to a simple repetitive block structure. Thus

$$C_k[1:P,](C_k + \sigma_k^2 I_{N_K P})^{-1} C_k[,1:P] = \frac{N_k}{\sigma_k^2}(A_k - A_k \times (I_p - Q^{-1}))A_k. \tag{17}$$

This means that only a single $P \times P$ matrix needs to be inverted and only two cases of multiplying a pair of $P \times P$ matrices must be performed rather than considering the inversion of a $N_k P \times N_k P$ matrix and its multiplication by a $P \times N_k P$ matrix and the result's multiplication by $N_k P \times P$ matrix.

We can now rewrite the posterior parameters as

$$m_k = \frac{N_k}{\sigma_k^2}(A_k - A_k \times (I_p - Q^{-1}))\bar{X}_k, \tag{18}$$

$$\tilde{C}_k = A_k - \frac{N_k}{\sigma_k^2}(A_k - A_k \times (I_p - Q^{-1}))A_k. \tag{19}$$

A.2 MDI: Derivation of $p(\phi_{(i,j)}|\cdot)$

A popular methods for performing inference on a Bayesian clustering method is to construct a Gibbs sampler. This uses the conditional densities of the model parameters. To derive these for MDI, we begin with the likelihood for the allocation of the nth individual in each of the V views, $c_n = (c_n^{(1)}, \ldots, c_n^{(V)})$ is

$$p(c_n^{(1)}, \ldots, c_n^{(V)}|\cdot) = \frac{S^{N-1}\exp(-SZ)}{(N-1)!} \times \prod_{n=1}^{N}\left(\prod_{v=1}^{V}\gamma^{(v)}_{c_n^{(v)}} \prod_{v=1}^{V-1}\prod_{w=v+1}^{V}(1 + \phi_{(v,w)}\mathbb{I}(c_n^{(v)} = c_n^{(w)})\right), \tag{20}$$

for N items being clustered using K components in each view, normalising constant Z, unnormalised component weights γ, information sharing parameter vector ϕ, and a strategic latent variable S that is introduced to induce closed posterior distributions for many of the variables. The conditional posterior distribution for these objects can be found in the supplementary material of [8]. In this supplement, the conditional distribution of the information sharing parameters, $\phi_{(i,j)}(i, j \in \{1, \ldots, V\} \subset \mathbb{N}, i < j)$ is stated as

$$\phi_{(i,j)} \sim Ga(\alpha, \beta), \tag{21}$$

where $Ga(a, b)$ is a Gamma distribution parameterised by a shape and a rate and

$$\alpha = 1 + \sum_{n=1}^{N} \mathbb{I}(c_n^{(i)} = c_n^{(j)}), \tag{22}$$

$$\beta = S \sum_{k_i=k_j=1}^{K} \sum_{k_1=1}^{K} \cdots \sum_{k_{i-1}=1}^{K} \sum_{k_{i+1}=1}^{K} \cdots \sum_{k_{j-1}=1}^{K} \sum_{k_{j+1}=1}^{K} \cdots \sum_{k_V=1}^{K} \prod_{v=1}^{V} \gamma_{k_v}^{(v)}$$
$$\times \prod_{v=1}^{V-1} \prod_{w=v+1;w\neq j}^{V} (1 + \phi_{(v,w)} \mathbb{I}(k_v = k_w)) \prod_{v=1}^{j-1} (1 + \phi_{(v,j)} \mathbb{I}(k_v = k_j)). \tag{23}$$

However, in 2017 Stephen Johnson, Daniel Henderson, and Richard Boys (private correspondence) noticed that this requires a correction which we derive here. Note that we assume $K_1 = K_2 = \cdots = K_V = K$, but this is not necessary and if it does not hold any quantities any occurrence of K can be replaced with the appropriate K_v for all $v = 1, \ldots, V$. To derive $p(\phi_{(i,j)}|\cdot)$, for some $i, j \in \{1, \ldots, V\}, i < j$, we derive the marginal likelihood of $\phi_{(i,j)}$ and then use this to find the closed form of the posterior distribution.

$$p(\phi_{(i,j)}|\cdot) \propto \exp\{-SZ\} \prod_{n=1}^{N} \left(\prod_{v=1}^{V-1} \prod_{w=v+1}^{V} (1 + \phi_{(v,w)} \mathbb{I}(c_n^{(v)} = c_n^{(w)}) \right) \tag{24}$$

we consider this in two parts, first the normalising constant in the exponential function and then the product on the right. The normalising constant, Z can be found by considering all possible combinations of states of the labels:

$$Z = \sum_{k_1=1}^{K} \cdots \sum_{k_V=1}^{K} \left(\prod_{v=1}^{V} \gamma_{k_v}^{(v)} \prod_{v=1}^{V-1} \prod_{w=v+1}^{V} (1 + \phi_{(v,w)} \mathbb{I}(c_n^{(v)} = c_n^{(w)}) \right). \tag{25}$$

Considering only the dependency of Z on $\phi_{(i,j)}$:

$$Z \propto \phi_{(i,j)} \sum_{k_i=k_j=1}^{K} \sum_{k_1=1}^{K} \cdots \sum_{k_{i-1}=1}^{K} \sum_{k_{i+1}=1}^{K} \cdots \sum_{k_{j-1}=1}^{K} \sum_{k_{j+1}=1}^{K} \cdots \sum_{k_V=1}^{K} \prod_{v=1}^{V} \gamma_{k_v}^{(v)}$$
$$\times \prod_{v=1}^{V-1} \prod_{w=v+1;w\neq j}^{V} (1 + \phi_{(v,w)} \mathbb{I}(k_v = k_w)) \prod_{v=1}^{j-1} (1 + \phi_{(v,j)} \mathbb{I}(k_v = k_j)). \tag{26}$$

The product simplifies as a function of $\phi_{(i,j)}$ to

$$\begin{aligned}
\prod_{n=1}^{N}\prod_{v=1}^{V-1}\prod_{w=v+1}^{V}(1+\phi_{(v,w)}\mathbb{I}(c_n^{(v)}=c_n^{(w)}) &\propto \prod_{n=1}^{N}(1+\phi_{(i,j)})\mathbb{I}(c_n^{(i)}=c_n^{(j)}) \\
&= (1+\phi_{(i,j)})^{\sum_{n=1}^{N}\mathbb{I}(c_n^{(i)}=c_n^{(j)})} \\
&= \sum_{r=0}^{\sum_{n=1}^{N}\mathbb{I}(c_n^{(i)}=c_n^{(j)})}\binom{\sum_{n=1}^{N}\mathbb{I}(c_n^{(i)}=c_n^{(j)})}{r}\phi_{(i,j)}^{r}, \qquad (27)
\end{aligned}$$

by the binomial theorem. $\sum_{n=1}^{N}\mathbb{I}(c_n^{(i)}=c_n^{(j)})$ is the number of items with the same label in both datasets. We denote this quantity by N_{ij} to reduce clutter.

Combining equations 26 and 27 yields:

$$\begin{aligned}
p(\phi_{(i,j)}|\cdot) \propto \exp\Bigg(&-\phi_{(i,j)}S\sum_{k_i=k_j=1}^{K}\sum_{k_1=1}^{K}\cdots\sum_{k_{i-1}=1}^{K}\sum_{k_{i+1}=1}^{K}\cdots\sum_{k_{j-1}=1}^{K}\sum_{k_{j+1}=1}^{K}\cdots\sum_{k_V=1}^{K}\prod_{v=1}^{V}\gamma_{k_v}^{(v)} \\
&\times\prod_{v=1}^{V-1}\prod_{w=v+1;v\neq j}^{V}(1+\phi_{(v,w)}\mathbb{I}(k_v=k_w))\prod_{v=1}^{j-1}(1+\phi_{(v,j)}\mathbb{I}(k_v=k_j))\Bigg) \\
&\times\sum_{r=0}^{N_{ij}}\binom{N_{ij}}{r}\phi_{(i,j)}^{r}. \qquad (28)
\end{aligned}$$

If we let $g(x;a,b):=\frac{b^a}{\Gamma(a)}x^{a-1}\exp(-bx)$ be the gamma density function, we can transform this to a mixture

of Gamma distributions:

$$\begin{aligned}
p(\phi_{(i,j)}|\cdot) \propto & \Bigg(S \sum_{k_i=k_j=1}^{K} \sum_{k_1=1}^{K} \cdots \sum_{k_{i-1}=1}^{K} \sum_{k_{i+1}=1}^{K} \cdots \sum_{k_{j-1}=1}^{K} \sum_{k_{j+1}=1}^{K} \cdots \sum_{k_V=1}^{K} \prod_{v=1}^{V} \gamma_{k_v}^{(v)} \\
& \times \prod_{v=1}^{V-1} \prod_{w=v+1;w\neq j}^{V} (1+\phi_{(v,w)}\mathbb{I}(k_v=k_w)) \prod_{v=1}^{j-1} (1+\phi_{(v,j)}\mathbb{I}(k_v=k_j)) \Bigg)^{r+1} \Gamma(r+1)^{-1} \\
& \times \Bigg(S \sum_{k_i=k_j=1}^{K} \sum_{k_1=1}^{K} \cdots \sum_{k_{i-1}=1}^{K} \sum_{k_{i+1}=1}^{K} \cdots \sum_{k_{j-1}=1}^{K} \sum_{k_{j+1}=1}^{K} \cdots \sum_{k_V=1}^{K} \prod_{v=1}^{V} \gamma_{k_v}^{(v)} \\
& \times \prod_{v=1}^{V-1} \prod_{w=v+1;w\neq j}^{V} (1+\phi_{(v,w)}\mathbb{I}(k_v=k_w)) \prod_{v=1}^{j-1} (1+\phi_{(v,j)}\mathbb{I}(k_v=k_j)) \Bigg)^{-(r+1)} \Gamma(r+1) \\
& \times \exp\Bigg(-\phi_{(i,j)} S \sum_{k_i=k_j=1}^{K} \sum_{k_1=1}^{K} \cdots \sum_{k_{i-1}=1}^{K} \sum_{k_{i+1}=1}^{K} \cdots \sum_{k_{j-1}=1}^{K} \sum_{k_{j+1}=1}^{K} \cdots \sum_{k_V=1}^{K} \prod_{v=1}^{V} \gamma_{k_v}^{(v)} \\
& \times \prod_{v=1}^{V-1} \prod_{w=v+1;w\neq j}^{V} (1+\phi_{(v,w)}\mathbb{I}(k_v=k_w)) \prod_{v=1}^{j-1} (1+\phi_{(v,j)}\mathbb{I}(k_v=k_j)) \Bigg) \\
& \times \sum_{r=0}^{N_{ij}} \binom{N_{ij}}{r} \phi_{(i,j)}^{r}, \qquad (29) \\
= & \sum_{r=0}^{N_{ij}} \binom{N_{ij}}{r} \frac{\Gamma(r+1)}{\beta^{r+1}} g(\phi_{(i,j)}; r+1, \beta), \qquad (30)
\end{aligned}$$

where

$$\begin{aligned}
\beta = & S \sum_{k_i=k_j=1}^{K} \sum_{k_1=1}^{K} \cdots \sum_{k_{i-1}=1}^{K} \sum_{k_{i+1}=1}^{K} \cdots \sum_{k_{j-1}=1}^{K} \sum_{k_{j+1}=1}^{K} \cdots \sum_{k_V=1}^{K} \prod_{v=1}^{V} \gamma_{k_v}^{(v)} \\
& \times \prod_{v=1}^{V-1} \prod_{w=v+1;w\neq j}^{V} (1+\phi_{(v,w)}\mathbb{I}(k_v=k_w)) \prod_{v=1}^{j-1} (1+\phi_{(v,j)}\mathbb{I}(k_v=k_j)) \qquad (31)
\end{aligned}$$

is the same as in the derivation for this posterior in the original paper.

If we then introduce a gamma prior distribution over $\phi_{(i,j)}$, say

$$\phi_{(i,j)} \sim Ga(\alpha_0, \beta_0), \qquad (32)$$

then the posterior distribution becomes

$$p(\phi_{(i,j)}|\cdot) \propto \frac{\beta_0^{\alpha_0}}{\Gamma(\alpha_0)} \phi_{(i,j)}^{\alpha_0 - 1} \exp(-\beta_0 \phi_{(i,j)}) \exp(-\phi_{(i,j)}\beta) \sum_{r=0}^{N_{ij}} \binom{N_{ij}}{r} \phi_{(i,j)}^{r} \tag{33}$$

$$\propto \sum_{r=0}^{N_{ij}} \binom{N_{ij}}{r} \phi_{(i,j)}^{r+\alpha_0-1} \exp(-\phi_{(i,j)}(\beta + \beta_0)) \tag{34}$$

$$= \sum_{r=0}^{N_{ij}} \binom{N_{ij}}{r} g(\phi_{(i,j)}|r + \alpha_0, \beta + \beta_0)\Gamma(r + \alpha_0)/(\beta + \beta_0)^{(r+\alpha_0)}. \tag{35}$$

which is clearly a mixture of Gamma densities, which can be rewritten as

$$p(\phi_{ij}|\cdot) = \sum_{r=0}^{N_{ij}} \pi_r g(\phi_{ij}|r + \alpha_0, \beta + \beta_0), \tag{36}$$

where

$$\pi_r = \frac{\binom{N_{ij}}{r}\Gamma(r + \alpha_0)/(\beta + \beta_0)^{(r+\alpha_0)}}{\sum_{r=0}^{N_{ij}} \binom{N_{ij}}{r}\Gamma(r + \alpha_0)/(\beta + \beta_0)^{(r+\alpha_0)}}. \tag{37}$$

The component of this mixture with the largest expected value is the same as the quantity in equation 21. This correction means that the sampled $\phi_{(i,j)}$'s will be smaller on average than in the original implementations, resulting in slightly less weight being placed by the model on common or overlapping partitions across views than in previous implementations.

B Simulation study

Each scenario is defined by the density used to generate the clusters (the generative model) and by the ϕ vector ($\phi = (12, 8, 4)$ for our simulation study) which determines the similarity of the clustering structure across modalities. We first generate the labels indicating the cluster an item is generated from in each modality and then sample measurements based on this cluster's parameters. In every simulation, the first dataset is generated from six clusters, the second from seven clusters and the third from eight clusters. We

generate two hundred labels and data points in each datasets from this model:

$$p(c_n^{(v)} = k|\gamma, \phi) \propto \gamma_k \prod_{w=1;w\neq v}^{V} (1 + \phi_{v,w}), \tag{38}$$

$$(X_n^{(v)}|c_n^{(v)} = k) \sim f(\theta_k), \tag{39}$$

$$\phi = (12, 8, 4), \tag{40}$$

$$K = (6, 7, 8), \tag{41}$$

$$\gamma^{(1)} = [4.0, 7.0, 7.0, 5.5, 3.0, 5.5]^\top, \tag{42}$$

$$\gamma^{(2)} = [5.5, 4.0, 4.0, 4.0, 4.0, 4.0, 3.0]^\top, \tag{43}$$

$$\gamma^{(3)} = [6.0, 6.0, 5.0, 3.0, 5.5, 7.0, 4.0, 4.0]^\top. \tag{44}$$

There are fifteen measurements / feature for each data point (i.e., $N = 200, P = 15$ in each dataset in all simulations), and in the first dataset of each simulation we have 6 clusters, in the second 7 and the third 8. Within a generating seed the labels in each modality are the same across all scenarios and each dataset is informative of the others ($\phi = (12, 8, 4)$). We randomly sample an expected 30% of the labels in the first dataset to be observed (or considered as training data) in the semi-supervised model runs.

We consider three different generating models which give the scenarios their names. The first two are

$$\text{Scenario 1 (Gaussian): } (X_n^{(v)}|c_n^{(v)} = k) \sim \mathcal{N}(\mu_k, \Sigma_k), \tag{45}$$

$$\text{Scenario 2 (MVT): } (X_n^{(v)}|c_n^{(v)} = k) \sim t_{\eta_k}(\mu_k, \Sigma_k). \tag{46}$$

Scenario 3, the Log-Poisson case, is more complex.

$$(Y_{n,p}|c_n^{(v)} = k) \sim Poisson(\lambda_{k,p}), \tag{47}$$

$$\epsilon_{n,p} \sim \mathcal{N}(0, 1), \tag{48}$$

$$X_{n,p} = \log(1 + Y_{n,p}) + \epsilon_{n,p}. \tag{49}$$

We randomly permute the cluster parameters in each feature/measurement. In the case of Scenarios 1

and 2, the generating means and standard deviations are:

$$\mu^{(1)} = [-1.50, -1.25, 1.25, 0.50, -1.00, 1.00, 1.75]^\top \tag{50}$$

$$\mu^{(2)} = [-1.00, -0.50, -0.75, 0.00, 0.25, 0.50, 0.75, 0.50, 0.00]^\top, \tag{51}$$

$$\mu^{(3)} = [-1.00, -0.50, -1.25, -0.50, -0.25, -0.25, 0.25, 1.25, 1.25, 1.00, 0.50]^\top, \tag{52}$$

$$\sigma^{(1)} = [1.50, 1.25, 1.75, 2.25, 2.75, 1.50, 3.0]^\top, \tag{53}$$

$$\sigma^{(2)} = [0.5, 1.0, 0.5, 1.0, 0.5, 0.25, 1.25, 0.5, 1.00^\top \tag{54}$$

$$\sigma^{(3)} = [0.50, 0.75, 1.50, 1.00, 0.50, 0.75, 0.75, 0.50, 1.50, 1.00, 0.50]^\top. \tag{55}$$

For scenario 2 we have, in addition to these means and standard deviations, the following degrees of freedom:

$$\nu^{(1)} = [5, 10, 5, 5, 10, 5, 10]^\top, \tag{56}$$

$$\nu_k^{(2)} \sim \mathcal{U}\{5, 10\} \text{ independently for each } k = 1 \ldots, K_2, \tag{57}$$

$$\nu_k^{(3)} \sim \mathcal{U}\{5, 10\} \text{ independently for each } k = 1 \ldots, K_3. \tag{58}$$

In Scenario 3 we have:

$$\lambda^{(1)} = [5, 15, 25, 35, 30, 15, 40]^\top \qquad , \tag{59}$$

$$\lambda^{(2)} = [4, 13, 21, 26, 55, 43, 31, 28, 15]^\top, \tag{60}$$

$$\lambda^{(3)} = [4, 8, 44, 32, 16, 28, 55, 24, 12, 18, 28]^\top. \tag{61}$$

C *Toxoplasma gondii* model convergence

The initial model runs displayed poor mixing in the LOPIT data (see figure A A). To circumvent this issue we used consensus clustering [9]. We found that an ensemble composed of the 15,000th sample from 150 chains displayed stability (figure B). The non-monotonic behaviour seen in figure B A for the wider ensembles is due to the emergence of the shared signal between the 9,000 and 12,000 iterations in a majority of chains (note the mass shifting up the scale in the figure B C as the depth increases). The consensus matrices for this inference and a similar consensus clustering for an unsupervised mixture model run on the microarray data can be seen in figure A B; this also shows that the clusters driving the disagreement across individual chains have their membership uncertainty well-captured by consensus clustering. We also performed consensus clustering of a mixture model on the microarray data to investigate the impact of the joint modelling of the

views. We use the same consensus clustering algorithm of the 15,000th iteration from 150 different chains as in the integrative analysis except we had to increase the number of components modelled to 300 as at 125 all available components were consistently occupied in MCMC samples in the initial model runs using the smaller number of components. This inference resulted in a much sparser consensus matrix than the MDI inference (see figure A B). The point estimates arising from these (defined using the `salso` method from the `salso` R package [10]) came to 47 clusters for MDI and 287 from the mixture model.

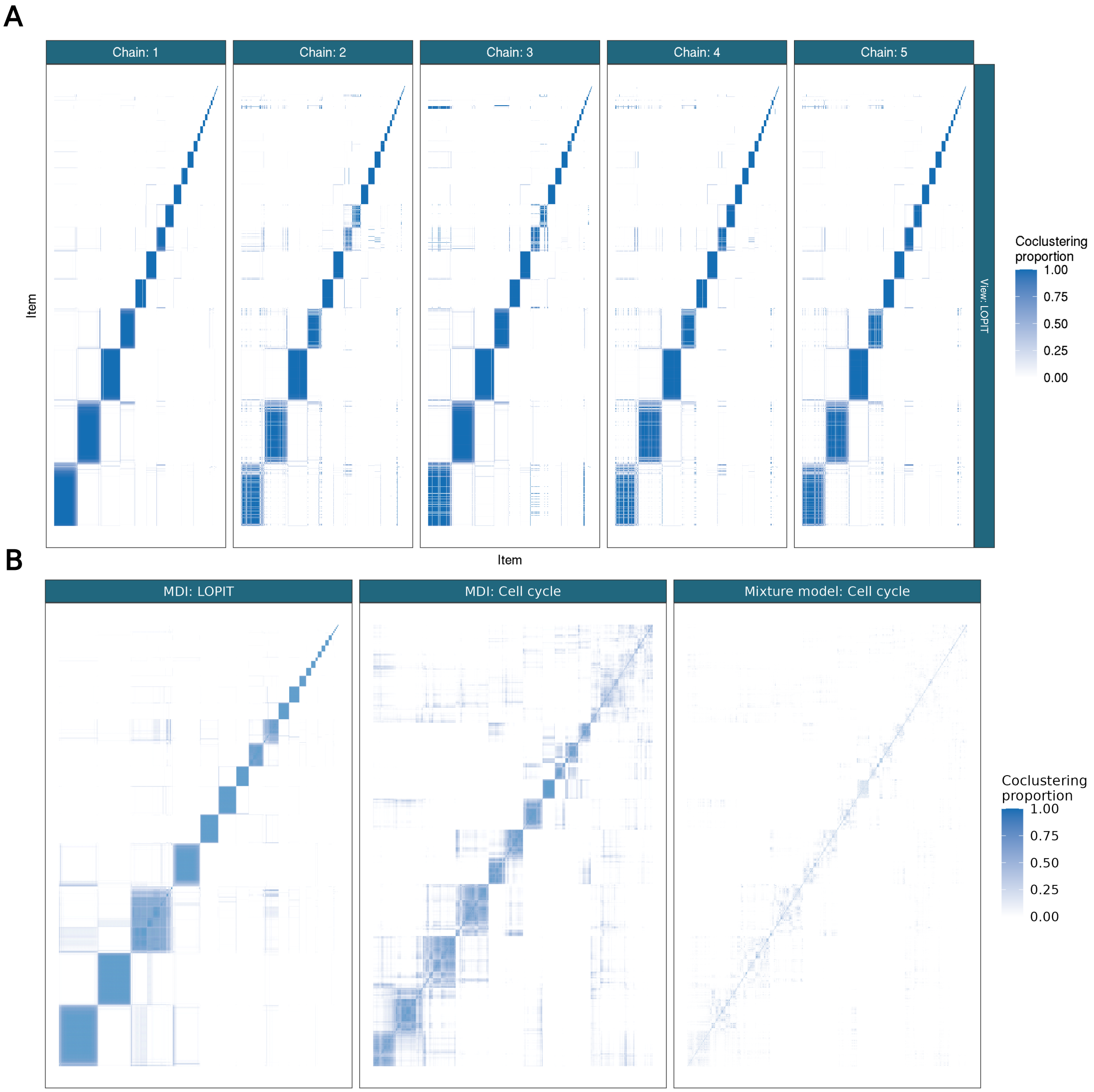

Figure A: A. PSMs for the LOPIT view of 5 chains of MDI run for 30,000 iterations with common row and column ordering (defined by chain 1). B. Consensus matrices for MDI in LOPIT and the cell-cycle microarray view and an unsupervised mixture model in the cell-cycle microarray data. The clusters that appear to be the cause of the poor mixing in the individual chains are highlighted with orange boxes in the LOPIT view.

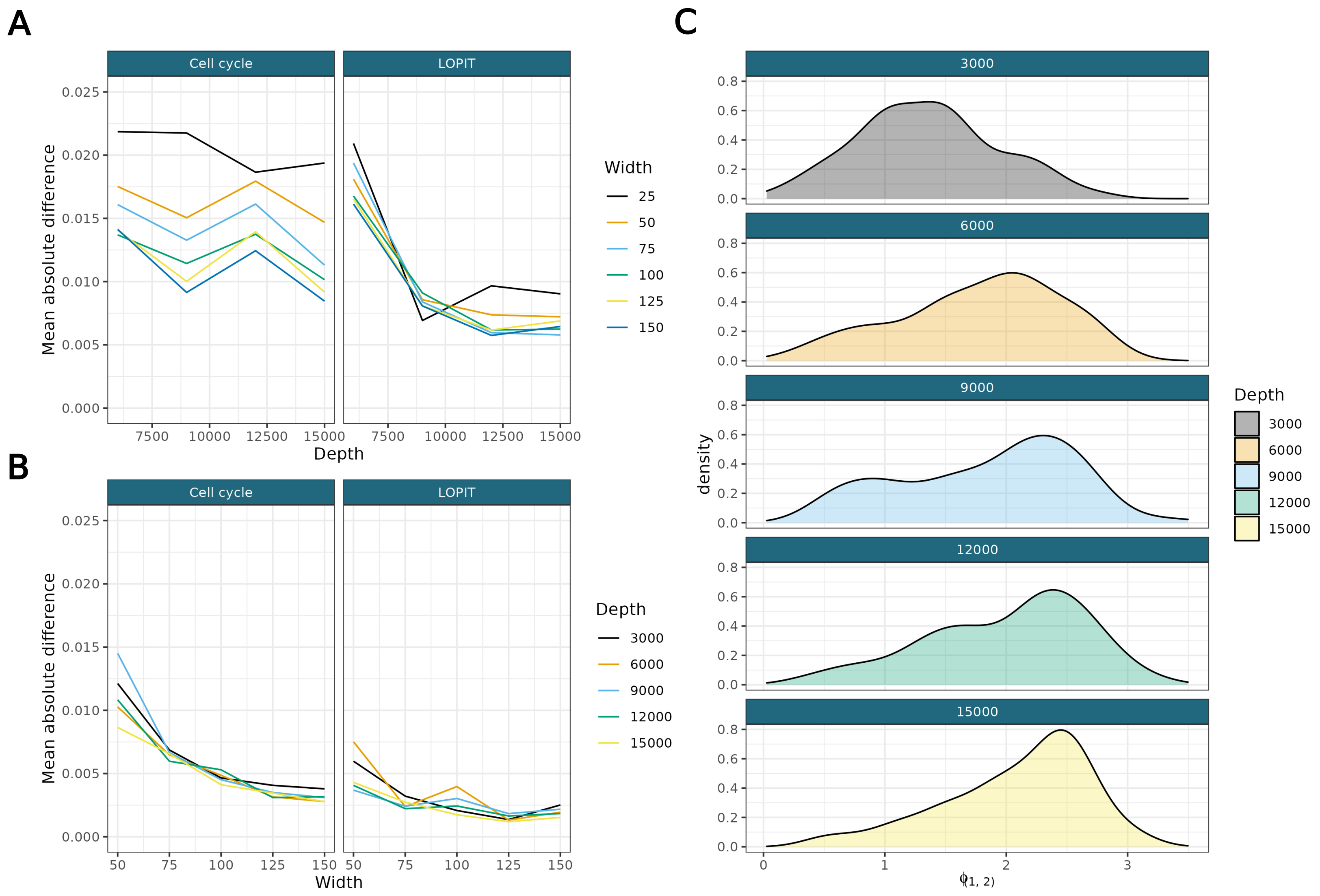

Figure B: A-B) Mean absolute difference between consensus matrices of increasing depth (A) and width (B). C) The sampled $\phi_{(1,2)}$ density for increasing depths.

References

[1] Williams CK, Rasmussen CE. Gaussian processes for machine learning. vol. 2. MIT press Cambridge, MA; 2006.

[2] Quinonero-Candela J, Rasmussen CE. A unifying view of sparse approximate Gaussian process regression. The Journal of Machine Learning Research. 2005;6:1939–1959.

[3] Banerjee A, Dunson DB, Tokdar ST. Efficient Gaussian process regression for large datasets. Biometrika. 2012;100(1):75–89. doi:10.1093/biomet/ass068.

[4] Liu H, Ong YS, Shen X, Cai J. When Gaussian Process Meets Big Data: A Review of Scalable GPs. IEEE Transactions on Neural Networks and Learning Systems. 2020;31(11):4405–4423. doi:10.1109/TNNLS.2019.2957109.

[5] Zhang Y, Leithead WE, Leith DJ. Time-series Gaussian Process Regression Based on Toeplitz Computation of O(N2) Operations and O(N)-level Storage. In: Proceedings of the 44th IEEE Conference on Decision and Control; 2005. p. 3711–3716.

[6] Golub GH, Van Loan CF. Matrix computations. John Hopkins University press; 2013.

[7] Crook OM, Lilley KS, Gatto L, Kirk PDW. Semi-supervised nonparametric Bayesian modelling of spatial proteomics. The Annals of Applied Statistics. 2022;16(4):2554 – 2576. doi:10.1214/22-AOAS1603.

[8] Kirk P, Griffin JE, Savage RS, Ghahramani Z, Wild DL. Bayesian correlated clustering to integrate multiple datasets. Bioinformatics. 2012;28(24):3290–3297.

[9] Coleman S, Kirk PDW, Wallace C. Consensus clustering for Bayesian mixture models. BMC Bioinformatics. 2022;23(1):290. doi:10.1186/s12859-022-04830-8.

[10] Dahl DB, Johnson DJ, Müller P. Search Algorithms and Loss Functions for Bayesian Clustering. Journal of Computational and Graphical Statistics. 2022;31(4):1189–1201. doi:10.1080/10618600.2022.2069779.
