# Supplementary material for: Semi-supervised Bayesian integration of multiple spatial proteomics datasets
Source: PLoS Comput Biol. 2025 Dec 15;21(12):e1013799. doi: 10.1371/journal.pcbi.1013799 (PMC12721539; doi:10.1371/journal.pcbi.1013799)
Supplement: Attachment — Submitted filename: Response to Reviewers.pdf [file pcbi.1013799.s002.pdf]

Response to Reviewers

We thank the reviewers for their thoughtful and constructive feedback, which has helped us improve the quality and clarity of our manuscript. Below, we provide a detailed, point-by-point response to all comments.

Reviewer 1:

The manuscript presents an innovative and well-supported approach to integrating spatial proteomics datasets using Bayesian models. The methodology is rigorous, and the results are compelling. Minor revisions to improve clarity, provide additional justification for certain methodological choices, and better contextualize findings in the broader field would strengthen the manuscript significantly.

We have endeavoured to add this context by the additions to the manuscript as detailed below. Each addition is paired with the specific request of the reviewer.

Abstract
Line 20-21:
The statement "existing approaches... do not quantify uncertainty" is strong. Consider specifying which methods lack this feature to prevent overgeneralization.

existing approaches for integrative analyses of spatial proteomics datasets, such as concatenation-based methods and transfer learning approaches like KNN-TL, are limited in the types of data they can integrate and do not quantify uncertainty in their predictions

Ensure "semi-supervised Bayesian approach" is briefly explained for a broader audience.

Here we propose a semi-supervised Bayesian (wherein model parameters are inferred from both labeled marker proteins and unlabeled data while quantifying prediction uncertainty) approach to integrate spatial proteomics datasets with other data sources

Line 24: What types of the data?

We demonstrate our approach outperforms other transfer-learning methods and has greater flexibility in the data it can model - including categorical annotations (e.g., Gene Ontology terms), continuous measurements (e.g., protein expression), and temporal profiles (e.g., time-course expression data).

Introduction
Lines 78–88: Consider restructuring the paragraph that discusses multi-omic integration challenges to emphasize why Bayesian methods are particularly suited for this task.
A direct comparison between past integrative methods and the proposed approach would help contextualize the contribution.

More generally, ’omic measurements can give us an integrated view on the molecular mechanisms of biological processes [34]. However, integrating diverse datasets (sometimes

referred to as modalities in the machine learning literature) is challenging because the different measurement processes generate heterogeneous data structures and each method has its own set of limitations.

Current integrative approaches have significant limitations for spatial proteomics applications. Simply concatenating the datasets assumes each dataset contributes equal information towards inference and ignores dataset specific variability.

Despite the successes of multi-omic data integration [35], most methods are unsupervised and tend to infer ~~so-called~~ latent factors that explain variation in the data; that is, a de-convolution of the dataset into a low-dimensional representation [36, 37] or to identify shared clusters [38– 40], but cannot leverage the valuable marker protein annotations available in spatial proteomics experiments. Transfer learning approaches like k-nearest neighbors [89] can incorporate auxiliary data but provide point estimates without uncertainty quantification and are limited to pairwise dataset comparisons.

Bayesian methods are particularly well-suited to address these limitations because they provide a principled framework for: (1) incorporating prior biological knowledge through marker proteins in a semi-supervised setting, (2) quantifying uncertainty in protein localisation predictions, (3) learning the degree of information sharing between datasets rather than assuming it, and (4) handling heterogeneous data types through modality-specific probability models [42]. Here, we perform integrative analysis of MS-based spatial proteomics data with other datasets (e.g. Gene Ontology (GO) annotations and Gene-expression datasets) as they contain powerful signals that may uncover subcellular regulation of processes that are not apparent from considering another dataset in isolation. Furthermore, the signal in one MS-based spatial proteomics dataset may be boosted by considering multiple datasets simultaneously. More generally, we wish to jointly model structure across datasets and uncover shared clusters, groups or regulation while properly accounting for uncertainty and dataset-specific characteristics.

42. Gelman A, Carlin JB, Stern HS, Dunson DB, Vehtari A, Rubin DB. Bayesian Data Analysis. 3rd ed. Chapman Hall/CRC Texts in Statistical Science Series. Boca Raton, Florida: CRC; 2013. Available from: https://stat.columbia.edu/~gelman/book/.

Lines 95–97: The sentence “Our approach is applicable beyond MS-based spatial proteomics...” is strong but could be supported with an example outside proteomics.

Our approach is applicable beyond MS-based spatial proteomics to general semi-supervised integrative tasks (such as the integration of clinical measurements with genomic data in personalized medicine applications)

Materials and Methods

The section introducing Gaussian mixture models (GMM) is mathematically rigorous but slightly dense.

Equations (6)–(9) are helpful, but it would be beneficial to provide a brief practical interpretation of each parameter.

In terms of the hierarchical model this is:

$$X_n | c_n, \theta \sim F(\theta_{c_n}), \quad (6)$$

$$c_n | \pi \sim \text{Categorical}(\pi_1, \ldots, \pi_K), \quad (7)$$

$$\pi_1, \ldots, \pi_K \sim \text{Dirichlet}(\alpha/K, \ldots, \alpha/K), \quad (8)$$

$$\theta_k \sim G(0), \quad (9)$$

where F is the appropriate distribution (e.g., Gaussian, Categorical, etc.) and G(0) is some prior over the component parameters. In practice, θ_k represents the parameters characterizing each cellular compartment (e.g., mean protein abundance profiles), π captures the relative sizes of compartments, and c_n indicates which compartment each protein belongs to. The hierarchical structure allows uncertainty to propagate through all levels of the model.

lines 177–197
could include a brief comparison to other integration models or simpler methods into the introduction part.

Figure 3 conveys important insights, but the differences between semi-supervised MDI and overfitted semi-supervised MDI should be better explained.

To make our intention with the simulation study more explicit we have added to the introduction paragraph where we state which methods we compare.

We fit five different models in each simulation. These are distinguished with the information available to them in the first dataset and whether they jointly model the datasets (i.e., MDI) or model each dataset separately using a mixture model, and if the model has access to class labels and the true number of classes present (e.g., when the number of subcellular niches is unknown). This comparison allows us to isolate the benefits of: (1) integrative vs. independent modeling, (2) supervised vs. unsupervised learning, and (3) known vs. inferred cluster numbers. In all cases datasets 2 and 3 are unsupervised and the number of clusters present is inferred. For the overfitted approaches, we use the overfitted mixture model framework of [63], which includes more mixture components than expected and allows the data to determine the effective number of clusters through Bayesian model selection. This creates two variants of semi-supervised MDI: (1) semi-supervised MDI where the true number of subcellular compartments is provided as prior knowledge, and (2) overfitted semi-supervised MDI where this number must be inferred from the data alone. The overfitted approach is often relevant to real applications where the total number of subcellular niches may be unknown, while the standard approach provides an upper bound on performance when complete prior knowledge is available.

- Unsupervised MDI: MDI with no observed labels and the number of clusters unknown,
- Overfitted semi-supervised MDI: MDI with known labels, but the true number of clusters is hidden,
- Semi-supervised MDI: MDI with both observed labels and the true number of clusters known,

- Overfitted semi-supervised mixture model: mixture model with known labels, but the true number of clusters is hidden,
- Semi-supervised mixture model: mixture model with both observed labels and the true number of clusters known,

63. Judith Rousseau and Kerrie Mengersen. Asymptotic behaviour of the posterior distribution in overfitted mixture models. Journal of the Royal Statistical Society: Series B (Statistical Methodology), 73(5):689–710, 2011.

Results
Simulation Study
The explanation of different generative models (Gaussian, MVT, Log-Poisson) is well-structured, but an intuitive justification for their selection (e.g., why these specific distributions?) would be helpful.

We consider three different generating models which give the scenarios their names. The first two are

Scenario 1 (Gaussian): $(X(v)n\ |c(v)n = k) \sim N\ (\mu k, \Sigma k)$, (33)

Scenario 2 (MVT): $(X(v)n\ |c(v)n = k) \sim t\eta k\ (\mu k, \Sigma k)$. (34)

Scenario 3, the Log-Poisson case, is more complex.

$(Yn,p|c(v)\ n = k) \sim P\ oisson(\lambda k,p)$, (35)

$n,p \sim N\ (0, 1)$, (36)

$Xn,p = \log\ (1 + Yn,p) + n,p$. (37)

This is inspired by the simulation study of Chandra et al. [91], but the Gaussian noise is added after the log-transform to ensure this transform is always possible. Each cluster strongly deviates from Gaussian in this case. An example of the simulated data is shown in figure 1 B. These distributions were selected to test robustness under different model assumptions: Gaussian represents ideal conditions matching model assumptions (often assumed for gene co-expression data), MVT tests heavy-tailed outlier scenarios common in proteomics, and Log-Poisson evaluates performance under severe distributional misspecification (based on log-normalization of count data such as RNA-seq). A full description of the generating mechanisms and choice of parameters to differentiate the datasets are given in S.2 of the supplementary material.

Figure 3 conveys important insights, but the differences between semi-supervised MDI and overfitted semi-supervised MDI should be better explained.

We have attempted to address this comment in the material & methods section introducing the simulation and models used (above) and we have done a significant rewrite of this section to improve clarity and increase relevance to a non-statistical audience as well as the caption of figure 3.

The predictive performance of the methods is shown in figure 3. ~~This figure demonstrates that, in the examples considered, knowing the true number of generating clusters in the semi-supervised methods is not better than estimating the number of clusters.~~ Semi-supervised MDI consistently outperforms all other approaches across datasets and scenarios, demonstrating the value of both integrative modeling and leveraging marker protein information. Notably, the overfitted semi-supervised MDI (which infers cluster number from data) performs as well as or better than the version with known cluster numbers, indicating robust automatic cluster detection under these simulation conditions. However, we note that in these examples the observed labels are sampled completely at random from the class, and that all classes are observed, enabling the overfitted mixture models to estimate K accurately and that if this was not the case, e.g., ~~However, if~~ the sampling of the observed labels was biased in some way, then performance might decline ~~or the choice of density used to model the classes was more severely misspecified then the overfitted model might need to use additional components to have a better fit. Investigating this further might be interesting, but it is reassuring that when the model is misspecified here, as in the MVT and Log-Poisson scenario, that it is providing a point estimate partition similar to the ground truth. In fact, it appears to have a mild advantage over the model with K known. We suspect that this is due to additional components capturing observations not well described by the Gaussian density describing their class of origin rather then attempting to fit all of the points into a smaller set of Gaussian densities.~~

The benefits of integration are most pronounced under model misspecification. In the Log-Poisson scenario, where the Gaussian modeling assumptions are severely violated, semi-supervised MDI maintains superior performance while mixture models deteriorate. This robustness stems from two factors: (1) the ϕ parameter in MDI reduces reliance on distributional assumptions by upweighting cluster assignments based on cross-dataset consistency, and (2) semi-supervised learning anchors predictions using marker proteins rather than relying solely on distributional fit.

Unsupervised MDI performs surprisingly well, often matching semi-supervised mixture models even without access to marker proteins (similar median performance across scenarios). This highlights the power of leveraging shared structure across datasets—information from complementary modalities can compensate for lack of labeled examples. However, when both integration and supervision are available (semi-supervised MDI), performance is consistently optimal across all scenarios.

~~We can also see that semi-supervised MDI is the best performer across all datasets and scenarios, though it is matched by unsupervised MDI in the Gaussian and MVT scenarios, the scenarios where the distributional assumptions of the model are either correct or close to correct. More generally, MDI outperforms or matches the mixture models. Unsupervised MDI frequently matches the semi-supervised mixture models in the first modality, with similar median performance across scenarios.~~

~~Unsurprisingly, semi-supervised MDI, which uses the the most information available to us by considering shared structure across datasets and the observed labels, performs the best in all datasets in the Log-Poisson scenario where the density choice is most strongly misspecified. The relative weight of the density likelihood in defining allocations is smaller in MDI than in the mixture model as the component weights are upweighted by the~~ ~~ϕ~~

~~parameter. Furthermore, in the first modality some labels are identified and thus the model has less reliance on the distributional assumptions than an unsupervised model or independent mixture models. Thus we would expect semi-supervised MDI to be more robust to model misspecification than the other methods investigated here, and it is reassuring to see this borne out.~~

The amended caption of figure 3:

Fig 3. Predictive performance of methods in the simulation study. Semi-supervised MDI consistently outperforms other approaches across all scenarios, with overfitted semi-supervised MDI (which infers cluster number from data) performing as well as the standard version (with known cluster number). Horizontal facets are the different datasets, vertical facets are the different generative scenarios (Gaussian: ideal conditions; MVT: heavy-tailed data; Log-Poisson: severe model misspecification). The y-axis shows the different methods compared and the x-axis is the Adjusted Rand Index (ARI) between the inferred labels and the ground truth, where higher values indicate better performance. For fair comparison ~~I~~in the first modality, the ARI is calculated on the same set of test proteins across all methods, excluding the marker proteins used for training in semi-supervised approaches. Each boxplot summarizes 100 simulation replicates.

~~for the same set of unknown labels even in the case of unsupervised MDI where the known labels are not used in the inference to make the comparison more appropriate. Each boxplot is composed of 100 points, the ARI between the clustering point estimate for a given model in a given dataset; e.g., the yellow box plot in the first facet is composed of the ARI between the true and inferred partition for the test (or unobserved) labels in the first dataset from the 100 simulations for the Gaussian scenario.~~

Discussion

The limitations section is somewhat brief. Potential areas to mention: Computational costs of methods, including MCMC for large datasets (big O complexity). Challenges in parameter selection and prior specification.

We have attempted to include big O complexity and acknowledge more limitations of our method, though we are not entirely clear on what the reviewer intends by ‘parameter selection’ in this context.

Our method has some limitations. Firstly, Bayesian methods are computationally intensive;~~.~~ Our method has complexity O(DNKV) for D MCMC samples, N proteins, K clusters, and V datasets, plus $O(MKP^2)$ for updating covariance parameters. This becomes prohibitive for very large datasets, though users are provided with principled uncertainty quantification and the ability to integrate heterogeneous data types in return for this cost. Note that the consensus clustering approach we employ [83] significantly reduces computational burden by using many short chains rather than few long chains, avoiding the need for lengthy burn-in periods while capturing multiple-modes in the parameter space.

Secondly, whilst we have shown some robustness to model misspecification, our analysis is likely to be affected by gross misspecification of the likelihood. In the integrative case, the mis-specification of a model for a single dataset may have a negative impact on the

modelling of all other datasets, as the misspecification “leaks” from one dataset into another where there is no misspecification, corrupting the analysis. In this case, one might apply “cut-models” or adapt our approach using an appropriate likelihood.

Third, prior specification requires careful consideration. We attempt to use uninformative or weak priors (e.g., for similarity parameters ϕ) that allow the data to dominate inference, or empirical Bayes approaches (for component parameters) to encourage meaningful prior choices, but this does not remove the complexity and subjective choice of prior.

Reviewer 2:

This manuscript by Coleman et al. addresses the problem of finding the subcellular localization of proteins in spatial proteomics experiments. Specifically, “LOPIT” type mass spectrometry experiments involves separating cellular proteins into sub-cellular fractions through multi-step centrifugation, followed by quantification of each fraction’s TMT profile using MS. Each organelle would have a different profile due to different density. The computational task then is to find the latent sub cellular localization from the TMT profile and potentially estimate uncertainty. This is typically done using supervised or semi-supervised classification approaches.

Here, the authors propose a multiple dataset integration framework that incorporates gaussian process modeling to incorporate different types of auxiliary data (categorical, time-series, etc.). This allows spatial proteomics mass spectrometry data to be combined with other data types (such as prior literature annotations) to improve classification performance. This work expands on a series of prior papers from the authors. Most notably, Crook et al. PLoS Comp Biol 2018 which introduced the Bayesian mixture model approach to analyze TMT fraction profiles, and also introduced the outlier T-distribution to catch non-conforming data (TAGM approach). Crook et al. PLoS Comp Biol 2020 then extended this approach toward semi-supervised discovery of new cluster/localization. Crook et al. Annals of Applied Statistics 2022 introduced the Gaussian Process mixture modeling approach. Breckels et al. PLoS Comp Biol 2016 introduced the KNN-TL classifier to include GO terms in compartment assignment.

Overall this looks to be an excellent paper that addresses an important need. As the authors stated, the sub cellular localization of proteins is a major determinant of their function. How to determine the dynamic localization of proteins in an unbiased and context-specific manner remains an incompletely solved problem. The proposed approach here appears to be robust and well justified. The authors demonstrate performance using both simulated data and existing experimental data sets. The rationale and notations are well explained, and the manuscript is well written. Another major strength is the availability of an R package so other investigators can take advantage of this advance.

I have no problem recommending acceptance, and only have the following minor comments. These are not necessary for acceptance but the authors’ considerations.

Comments:

1. The primary goal of this work, as I understand it, or at least as laid out in the abstract/introduction, is to improve on the inference of protein sub cellular localization. This appears to have evolved somewhat as the manuscript progresses to the T. gondii results section. It would be nice to have more details on why the authors opted to incorporate the time-series data as opposed to other types of data (e.g., co-expression or interactome data), and intuitively how this may help with localization assignment. When the protein assignment changes this way (e.g., ERK7 or the dense granule proteins), does it indicate a change in actual sub cellular localization, or that different proteins within the same localization can take on different function or temporal behavior?

You raise an excellent point about our choice of transcriptomic data and interpretation of protein assignment changes. Our selection of cell cycle time-course gene expression data is based on the supposition that proteins which co-localize are more likely to share similar transcriptional programs; particularly in the case of organelles relating to invasion of human cells and the circumvention of immune response. The successful construction of these organelles (and thus, correct localization) involves coordinated temporal expression programs (Lou et al., 2024, Khelifa et al., 2021, Sakura et al., 2016). We should also emphasize that the reciprocal holds; that the modalities are mutually reinforcing and the expression clusters are more meaningful when jointly modelled with the LOPIT data. Specifically, when we use the standalone mixture model on this data the point estimate for the number of clusters is 453 in contrast to MDI which, using the same method of arriving at a point estimate, finds 47 clusters. Thus the ability to interpret the expression data is significantly improved, both due to the smaller number of clusters to interpret or merge but also due to their link to localization.

We used this data in the belief that proteins which co-localize will have a better-than-random co-clustering behaviour in the expression data, even if this is across multiple clusters. Thus we hope that for this poorly annotated organism we can use this additional data to derive more informed predictions of localization, and possibly uncover more high-resolution organization. The first applies most strongly in the example of boundary proteins that were only weakly identified as belonging to their previous organelle. For the latter, proteins which we find to have more similar spatial profiles and share transcriptional modules might be better considered as belonging to sub-niches; not necessarily entirely novel organelles but rather spatially distinct, more homogeneous parcels of organization within the current classification. For example, our analysis divides dense granule proteins into populations with different temporal expression patterns, suggesting functional specialization within the same spatial compartment. For ERK7, the low assignment probability in our analysis may reflect its known context-dependent localization properties (Back et al., 2020, O'Shaughnessy et al., 2023), where temporal regulation provides additional functional context beyond spatial information alone.

We should also emphasize that our MDI framework does not override strong spatial proteomics evidence based solely on gene expression patterns. The integration parameter ϕ is learned from the data to appropriately weight each modality. Proteins most likely to be reclassified are those showing strong temporal co-clustering with organelle-specific genes but lacking clear spatial proteomics signal.

Lou, J., Rezvani, Y., Arriojas, A. *et al.* Single cell expression and chromatin accessibility of the *Toxoplasma gondii* lytic cycle identifies AP2XII-8 as an essential ribosome regulon driver. *Nat Commun* **15**, 7419 (2024). https://doi.org/10.1038/s41467-024-51011-7

Khelifa, A.S., Guillen Sanchez, C., Lesage, K.M. *et al.* TgAP2IX-5 is a key transcriptional regulator of the asexual cell cycle division in *Toxoplasma gondii*. *Nat Commun* **12**, 116 (2021). https://doi.org/10.1038/s41467-020-20216-x

Sakura, T., Sindikubwabo, F., Oesterlin, L. *et al.* A Critical Role for *Toxoplasma gondii* Vacuolar Protein Sorting VPS9 in Secretory Organelle Biogenesis and Host Infection. *Sci Rep* **6**, 38842 (2016). https://doi.org/10.1038/srep38842

P.S. Back, W.J. O'Shaughnessy, A.S. Moon, P.S. Dewangan, X. Hu, J. Sha, J.A. Wohlschlegel, P.J. Bradley, & M.L. Reese, Ancient MAPK ERK7 is regulated by an unusual inhibitory scaffold required for *Toxoplasma* apical complex biogenesis, *Proc. Natl. Acad. Sci. U.S.A.* 117 (22) 12164-12173, https://doi.org/10.1073/pnas.1921245117 (2020).

William J. O'Shaughnessy, Xiaoyu Hu, Sarah Ana Henriquez, Michael L. Reese, Toxoplasma ERK7 defends the apical complex from premature degradation, bioRxiv 2021.12.09.471932; doi: https://doi.org/10.1101/2021.12.09.471932

Lines 261-265

Motivated by the importance of these organisms and their complex cellular structure, we perform ~~an~~ multi-omic analysis of the model apicomplexan, T. gondii, combining hyperLOPIT data from Barylyuk et al. [31] and transcriptomic data for the cell-cycle over twelve hours of post-invasion parasite replication [109] (figure 2). ~~We expect that this cell cycle data can~~ We use cell cycle time-series gene expression data due to recent evidence that tightly co-ordinated transcriptional programs are essential for proper organelle (Sakura et al., 2016, Lou et al., 2024), and we expect this to reveal reveal the transcriptional programmes of the genes involved in organelle formation and the coexpression patterns of the secreted proteins (Khelifa et al., 2021). Additionally, our MDI framework does not force proteins with strong spatial proteomics evidence to relocalize based purely on temporal data - proteins most likely to benefit from integration are those with ambiguous spatial signals but strong temporal co-clustering with organelle-specific genes.

Line 415

…more in line with the timing of biogenesis of small granules within Cryptosporidium. Our analysis suggests that there is additional heterogeneity within the dense granules and, depending on the scientific question, it might be beneficial to consider more homogeneous sub-niches within these heterogeneous bodies.

Within the supplement we expand the section comparing performing inference using MDI compared to the standalone mixture model on the temporal data in section S.3:

We use the same consensus clustering algorithm of the 15,000th iteration from 150 different chains as in the integrative analysis except we had to increase the number of components modelled to 300 as at 125 all available components were consistently occupied in MCMC samples in the initial model runs using the smaller number of components. This inference resulted in a much sparser consensus matrix than the MDI inference (see figure S.1 B). The point estimates arising from these (defined using the `salso` method from the `salso` R package) came to 47 clusters for MDI and 287 from the mixture model.

Dahl, D. B., Johnson, D. J., & Müller, P. (2022). Search Algorithms and Loss Functions for Bayesian Clustering. *Journal of Computational and Graphical Statistics*, *31*(4), 1189–1201. https://doi.org/10.1080/10618600.2022.2069779

2. Since the AOAS 2022 paper already described the GP model in some detail, it would be nice to have more clarity on how the current method compares, and also include the semi-supervised GP model as comparison along with the TAGM mixture models in benchmarking (e.g., in Figures 3-5). Also as far as I know, previous methods from the authors already had the capacity to integrate multiple independent sets of LOPIT TMT data. To show the value of multi datatype integration, it would be nice to directly compare whether incorporating

We did consider using the semi-supervised GP model in the LOPIT modality as part of the integrative analysis (this is implemented as part of the R package), but it proved very computationally intensive - we would expect that the GP model would produce lower variance parameters for the organelle classes due to the (correct) assumption of spatial dependence in the neighbouring fraction measurements, but would not expect this to change the overall prediction of many proteins here.

Regarding integrating independent sets of LOPIT TMT, part of our intention is to show that we are capable of integrating more diverse modalities than LOPIT alone and also are not limited in how many we can integrate (except due to the available computational budget).

3. Did the authors investigate the effect of GO categorical annotation on difficult-to-localize outliers, e.g., if a protein is annotated to be in either the cytosol or the nucleus in GO or UniProt, would the performance gain in protein assignment reflect this by preferentially localizing a protein to those compartments? It would be interesting as well if the authors could comment on whether this integration can help the assignment of differential localization between the two annotated compartments.

We did include the GO annotation in the validation study and in the more complex datasets (Mouse and Human derived) the inclusion significantly improved the ability of the method to recapture the true localization as shown in figure 4.

4. I was not completely clear on how accuracy and other performance metrics were calculated in the validation study. Were the true positives used to calculate these metrics picked from holdout markers that were then not used to train the supervised/semi-supervised models? As far as I know training the TAGM mixture model requires 20-30% of proteins to be designated as markers and how the markers are chosen can have an effect on the classification outcome. Did the authors investigate whether the change in markers affected the TAGM mixture model and the MDI approach differently? (For instance, it is not clear how the use of GO annotations for integration affects marker selection. Is manual marker selection still required for the MDI method, or can literature annotation be used to skip manual markers?)

We created random splits of 30% of the protein localizations considered known (i.e., treated as marker proteins) and 70% unknown (i.e., to be inferred), selecting marker proteins uniformly at random from the full set, with the constraint that all organelles always had at least one marker protein.

We do not consider the wider problem of designing a marker set - as the reviewer suggests this is a key part of an analysis, but is beyond the remit of the model. Consider that we select markers poorly (e.g., a subset inconsistently localize to the organelle they are representing). This should have similar consequences as for the TAGM model (from the model's perspective these are "true" marker proteins), but if the orthogonal datasets are informative, how deeply it influences the final classification should be mitigated as long as some of the marker proteins are representative as we would expect more members of the organelle to co-cluster (possibly across multiple clusters) with the true marker proteins in the additional datasets.

MDI can be run unsupervised (i.e., no marker proteins), as shown in our simulation study. The most relevant scenario is probably the 'Log-Poisson' scenario where the model is misspecified; here the model can still provide meaningful insights, but we believe that the orthogonal data must be considered more carefully to ensure shared signal does relate to the inference of interest (here, co-localization); additionally it is possible that post-inference merging of clusters will be necessary (the model might learn more fine scale features than is of interest to the biologist).

5. Can the authors explain in greater details what the phi parameter is and how it is estimated, and how 12, 8, 4 were chosen for the simulation study?

Phi is the "information sharing" parameter. It reflects how strongly the discrete structure (i.e., the clusters, the classes) in a pair of datasets correlates. Thus in a case where we have 3 datasets, $\phi = (\phi_{\{1,2\}}, \phi_{\{1,3\}}, \phi_{\{2,3\}})$, i.e., there is a phi parameter between each pair of datasets. We chose decreasing degrees of dependence between the datasets (12 is quite strong but still some dataset unique signal; 4 is very weak with slightly more shared signal than purely at random) to reflect realistic combinations of datasets. In the MDI model, ϕ is inferred jointly with all other parameters - we do not a priori set this and have a weak Ga(1, 1) prior on each member of the vector.

6. A major strength here is the implementation of an R package but the Github of MDIr appears to contain little to no documentation. While the repository is not strictly part of the paper, I believe this should be fixed to make the paper more useful to the readers. Besides documentations and/or tutorial, it would also be useful to have more discussion on how users may apply their own data - e.g., computational requirements, what type of mass spec data is allowed, how to tune parameters, etc.

We have added a discussion of the computational cost to the discussion in terms of big O notation, and added several vignettes to the github repository (particularly expanding the README).

Our method has complexity O(DNKV) for D MCMC samples, N proteins, K clusters, and V datasets, plus $O(MKP^2)$ for updating covariance parameters.

7. It would be useful to have additional discussion on how robust the method is to data input (e.g., some existing LOPIT data are rather sparse with low mass spec depth, others may use suboptimal or misannotated markers, etc. etc.)

Due to the Bayesian nature of the method, low data quality should be reflected in higher uncertainty in the final posterior distributions, and misannotated markers should be partially offset by informative orthogonal data (as discussed above in our response to point 4), but if the data is low quality of too many marker proteins are misannotations, the method can only do so much - the inference, like in all statistical methods, is based on the assumptions (which in this case would include incorrect assumptions in the misannotations) and can only be mitigated so well. We have shown in the simulation study that we have some robustness, and we would note that this method is intended for modern or future datasets rather than addressing older data characteristics. We would note that if one has markers one doubts, one could initialize the clustering from them but not fix them in place (i.e., a user-defined initialization of an unsupervised approach). Additionally, if data is sparse (e.g., some proteins or genes lack a measurement for one feature or more features) the method can treat the missing data as another random variable to be inferred. Though none of the datasets in the manuscript had this problem, the R package has this functionality built in.

8. Some typos/formatting errors, e.g., line 37 time-course; supplement line 56 and 105, missing ref.

Thank you; we have corrected these. To be clear that the correspondence from Johnson, Henderson and Boys does not have a reference we have explicitly stated:

as observed by Stephen Johnson, Daniel Henderson, and Richard Boys in 2017 (private correspondence; see supplement section S.1.2).
